# Supplementary material for: Range of motion and between-measurement variation of spinal kinematics in sound horses at trot on the straight line and on the lunge
Source: PLoS One. 2020 Feb 25;15(2):e0222822. doi: 10.1371/journal.pone.0222822 (PMC7041811; doi:10.1371/journal.pone.0222822)
Supplement: S1 Fig — These data enable the evaluation of the amount and differences in variation. (DOCX) [file pone.0222822.s001.docx]

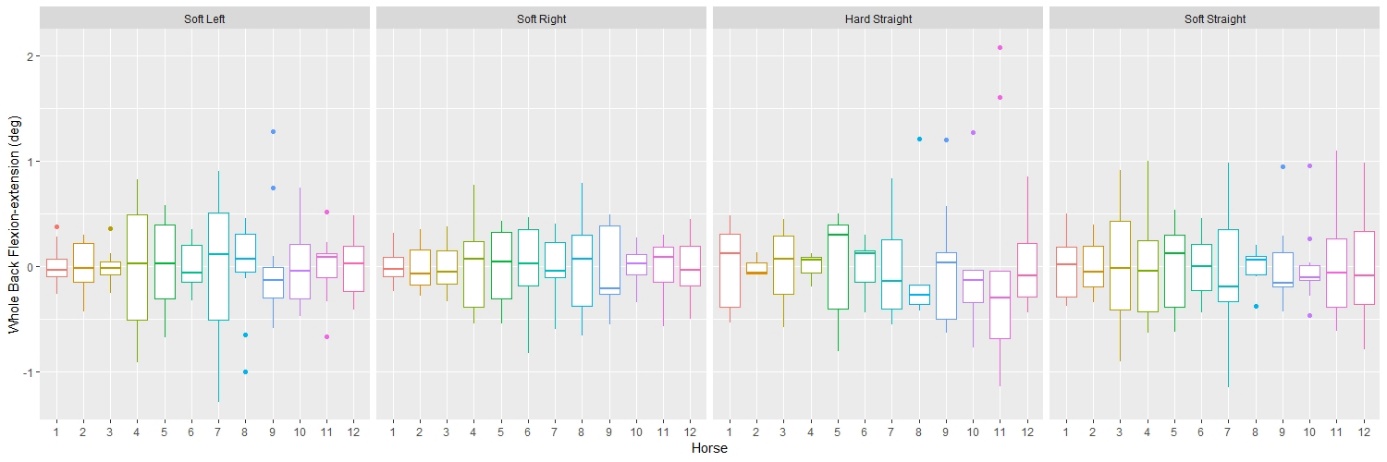

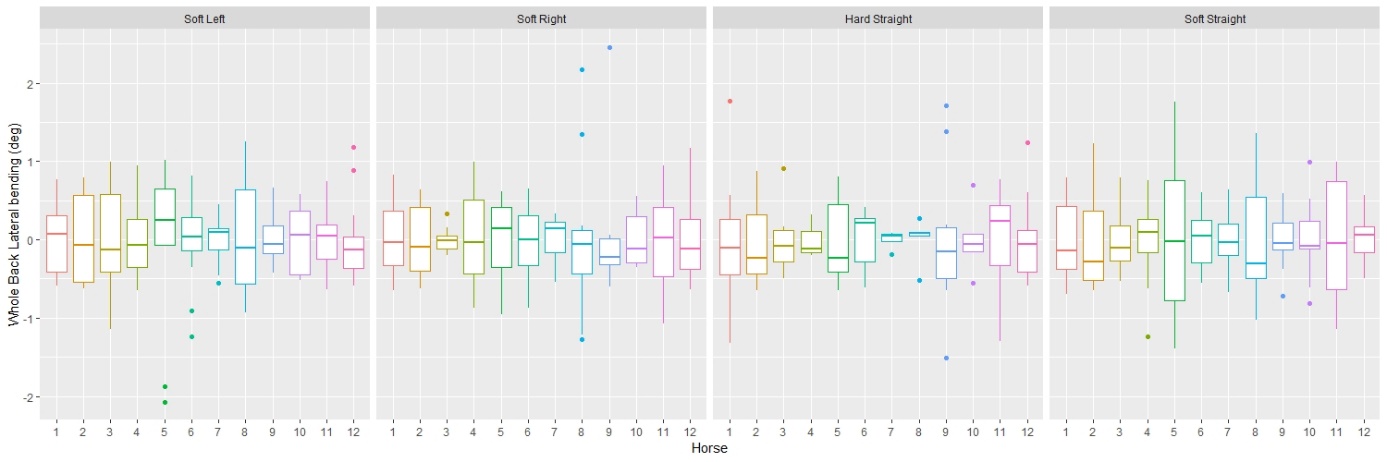

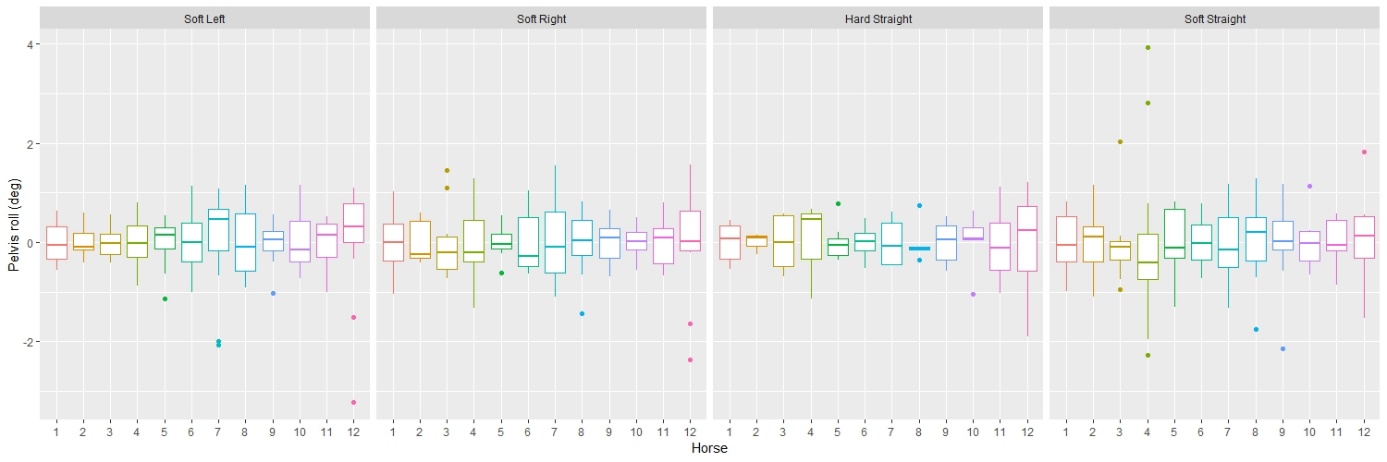

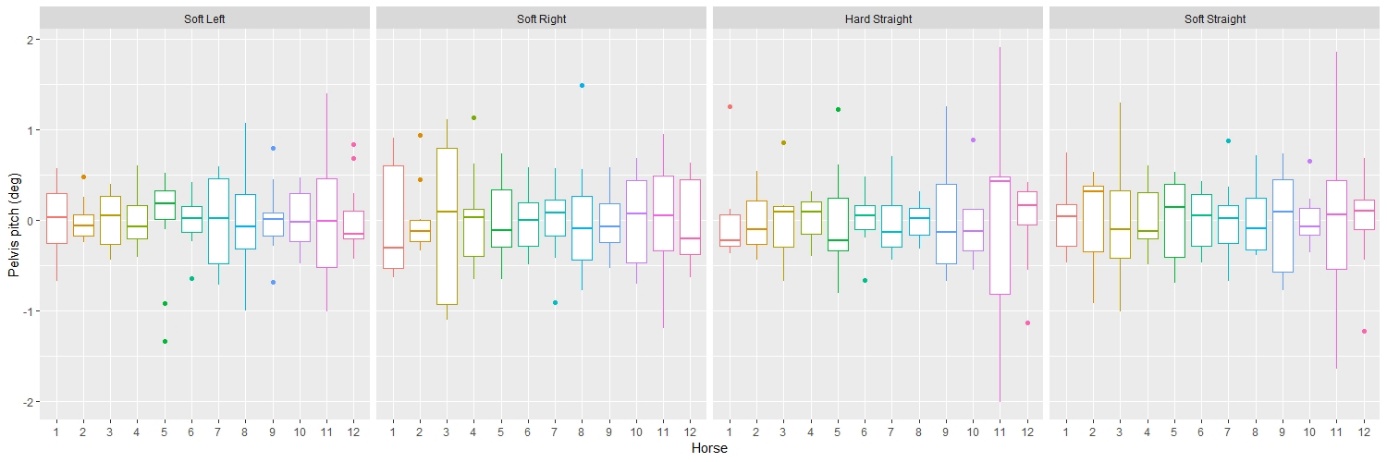

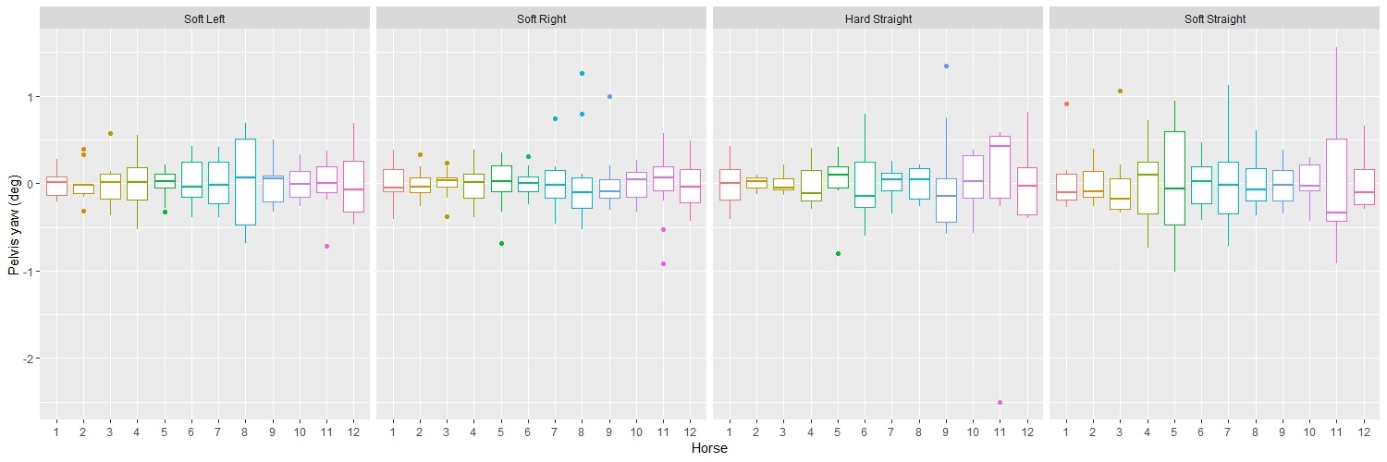

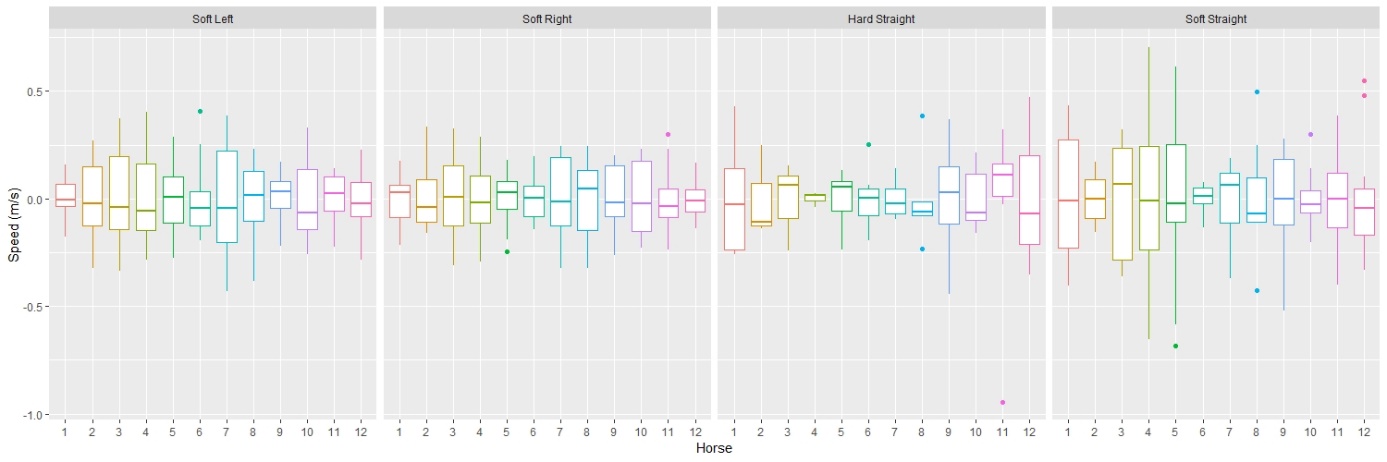

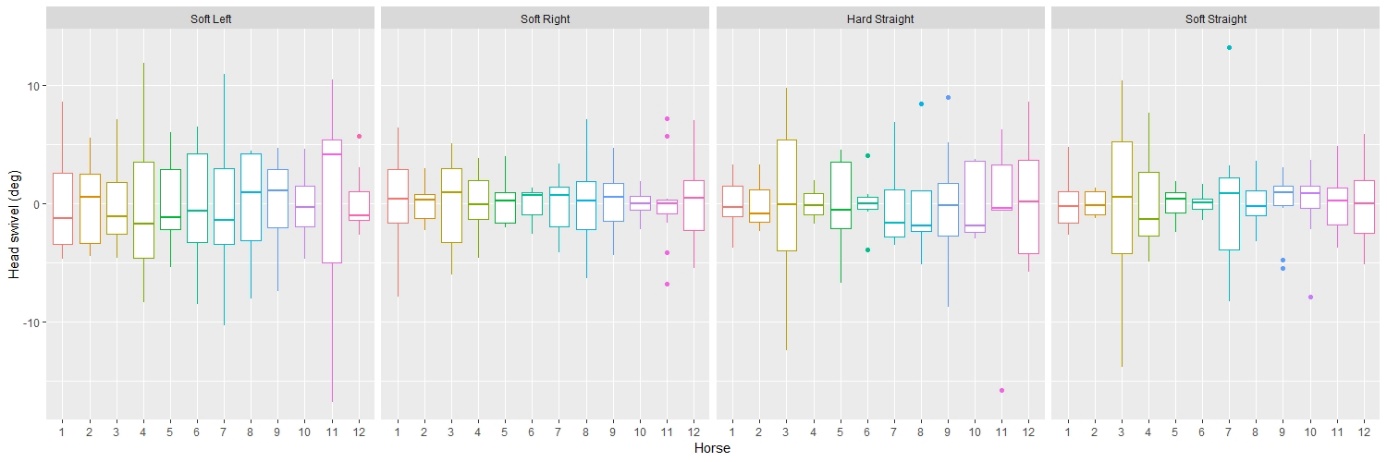

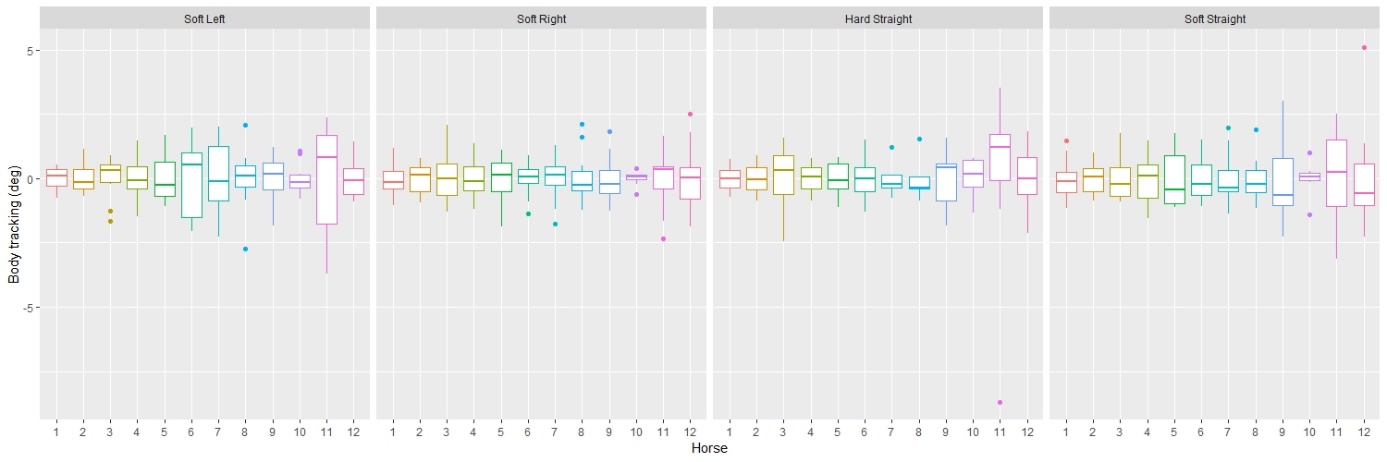


**S1 Fig. Between-measurement-variation (Offset adjusted data) per horse and per path over all measurements. Here given for the parameters ‘Whole Back Flexion-extension’, ‘Whole Back Lateral bending’, ‘Pelvis roll’, ‘Pelvis pitch’, ‘Pelvis yaw’, ‘Speed’, ‘Head swivel’ and ‘Body tracking’.** These data enable the evaluation of the amount and differences in variation.
