# Supplementary material for: Range of motion and between-measurement variation of spinal kinematics in sound horses at trot on the straight line and on the lunge
Source: PLoS One. 2020 Feb 25;15(2):e0222822. doi: 10.1371/journal.pone.0222822 (PMC7041811; doi:10.1371/journal.pone.0222822)
Supplement: S1 Table — *Horses were measured at different timepoints during the recheck (M11). ** M12 was done 5 minutes after M11. (DOCX) [file pone.0222822.s002.docx]

| **Day** | **Horse** | **M1&M6** | **M2&M7** | **M3&M8** | **M4&M9** | **M5&M10** | **M11** | **M12** |
| --- | --- | --- | --- | --- | --- | --- | --- | --- |
| **1** | 1 | 8:00 | 8:05 | 8:15 | 8:25 | 8:35 |  |  |
|  | 2 | 8:50 | 8:55 | 9:05 | 9:15 | 9:25 |  |  |
|  | 3 | 9:40 | 9:45 | 9:55 | 10:05 | 10:15 |  |  |
|  | 4 | 10:30 | 10:35 | 10:45 | 10:55 | 11:05 |  |  |
|  | 5 | 11:20 | 11:25 | 11:35 | 11:45 | 11:55 |  |  |
| **2** | 1 | 8:00 | 8:05 | 8:15 | 8:25 | 8:35 |  |  |
|  | 2 | 8:50 | 8:55 | 9:05 | 9:15 | 9:25 |  |  |
|  | 3 | 9:40 | 9:45 | 9:55 | 10:05 | 10:15 |  |  |
|  | 4 | 10:30 | 10:35 | 10:45 | 10:55 | 11:05 |  |  |
|  | 5 | 11:20 | 11:25 | 11:35 | 11:45 | 11:55 |  |  |
| **3** | 6 | 8:00 | 8:05 | 8:15 | 8:25 | 8:35 |  |  |
|  | 7 | 8:50 | 8:55 | 9:05 | 9:15 | 9:25 |  |  |
|  | 8 | 9:40 | 9:45 | 9:55 | 10:05 | 10:15 |  |  |
|  | 9 | 10:30 | 10:35 | 10:45 | 10:55 | 11:05 |  |  |
|  | 10 | 11:20 | 11:25 | 11:35 | 11:45 | 11:55 |  |  |
|  | 11 | 12:10 | 12:15 | 12:25 | 12:35 | 12:45 |  |  |
|  | 12 | 13:00 | 13:05 | 13:15 | 13:25 | 13:35 |  |  |
| **4** | 6 | 8:00 | 8:05 | 8:15 | 8:25 | 8:35 |  |  |
|  | 7 | 8:50 | 8:55 | 9:05 | 9:15 | 9:25 |  |  |
|  | 8 | 9:40 | 9:45 | 9:55 | 10:05 | 10:15 |  |  |
|  | 9 | 10:30 | 10:35 | 10:45 | 10:55 | 11:05 |  |  |
|  | 10 | 11:20 | 11:25 | 11:35 | 11:45 | 11:55 |  |  |
|  | 11 | 12:10 | 12:15 | 12:25 | 12:35 | 12:45 |  |  |
|  | 12 | 13:00 | 13:05 | 13:15 | 13:25 | 13:35 |  |  |
| **28-55** | 1-12 |  |  |  |  |  | 0* | +5 min.** |

**S1 Table. Time schedule of all measurements (M1-M12).** *Horses were measured at different timepoints during the last day (M11). ** M12 was done 5 minutes after M11.
