# Supplementary material for: Range of motion and between-measurement variation of spinal kinematics in sound horses at trot on the straight line and on the lunge
Source: PLoS One. 2020 Feb 25;15(2):e0222822. doi: 10.1371/journal.pone.0222822 (PMC7041811; doi:10.1371/journal.pone.0222822)
Supplement: S2 Table — Calculated arithmetic means of the predictions are shown in the last column. (DOCX) [file pone.0222822.s003.docx]

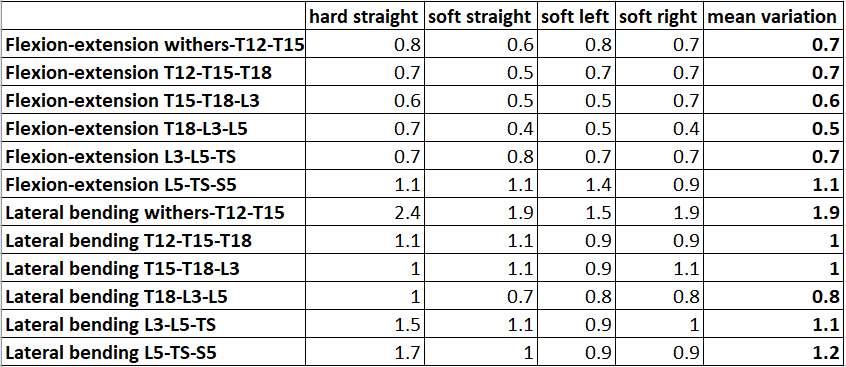


**S2 Table. Between-measurement-variation in degrees, given as the (absolute) prediction interval, per condition and per parameter. Calculated arithmetic means of the predictions are shown in the last column.**
