# Supplementary material for: Range of motion and between-measurement variation of spinal kinematics in sound horses at trot on the straight line and on the lunge
Source: PLoS One. 2020 Feb 25;15(2):e0222822. doi: 10.1371/journal.pone.0222822 (PMC7041811; doi:10.1371/journal.pone.0222822)
Supplement: S3 Table — Intercept = referenced level (day one, straight line, soft surface). Significance codes: 0 − < 0.001 ‘***’ 0.001 − < 0.01 ‘**’ 0.01 − < 0.05 ‘*’ 0.05 − < 0.1 ‘’. (DOCX) [file pone.0222822.s004.docx]

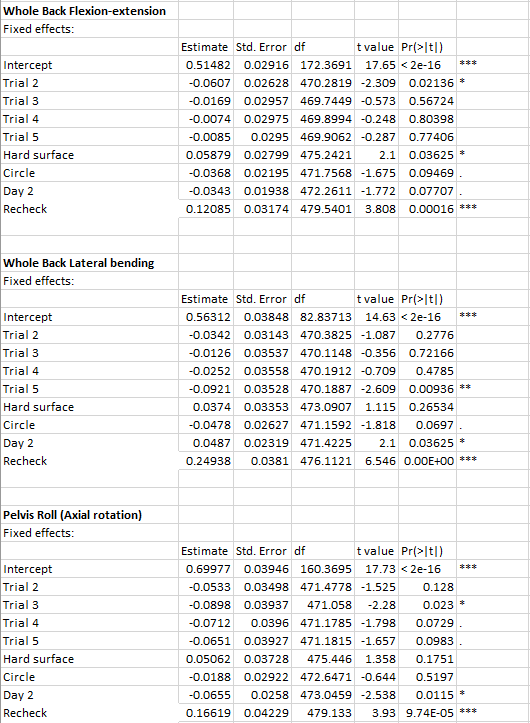

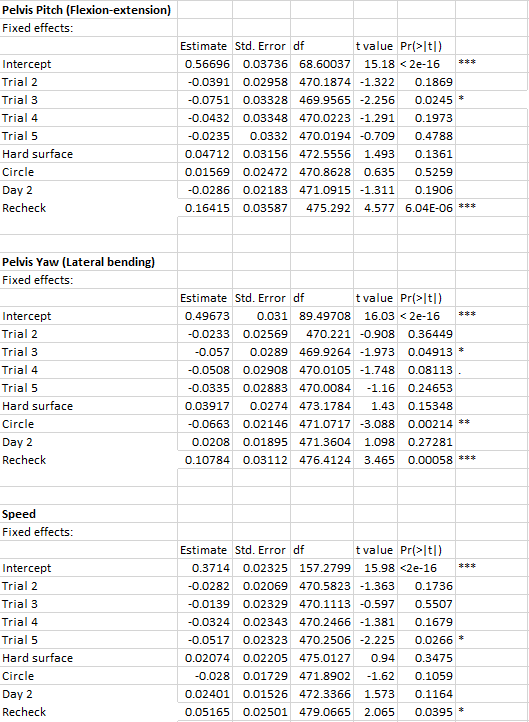

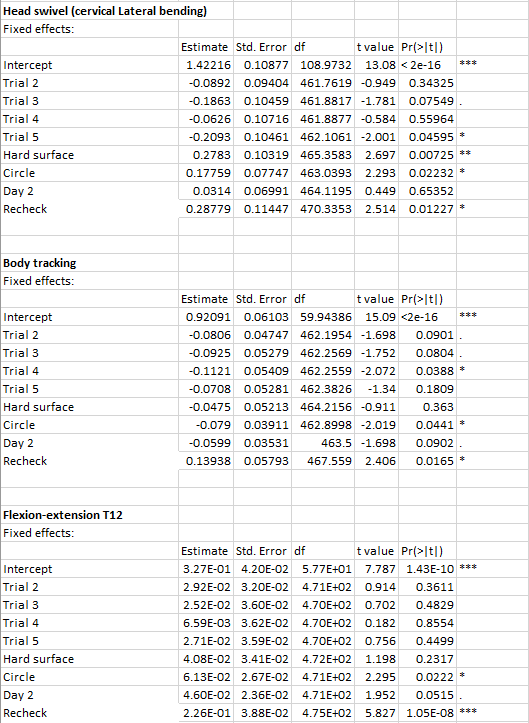

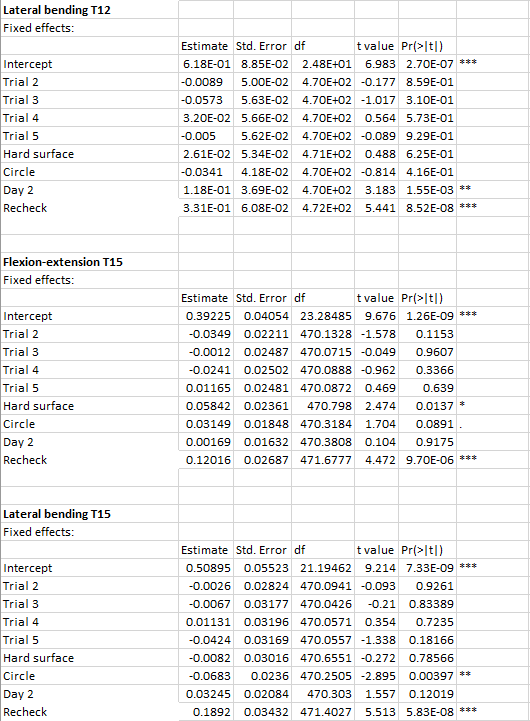

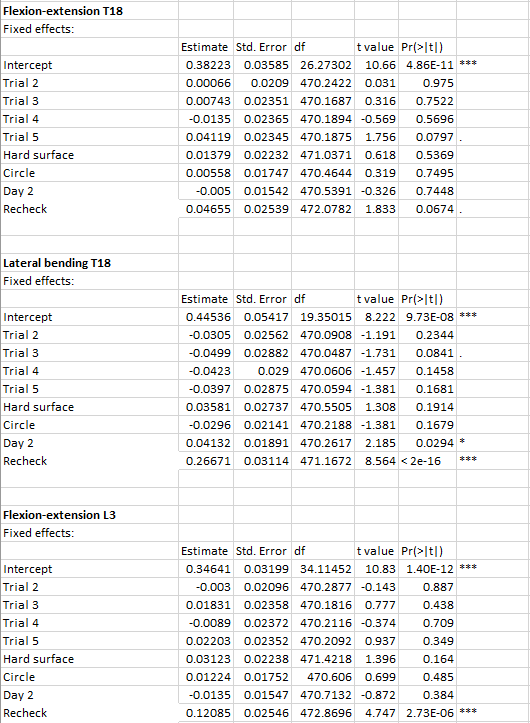

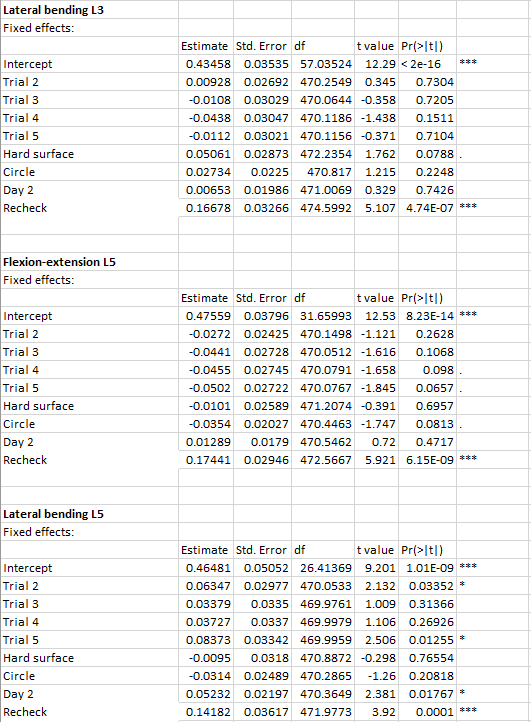

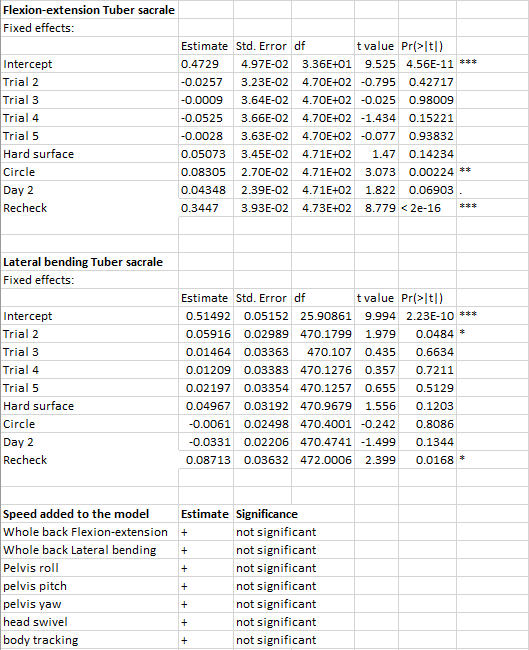


**S3 Table. Model estimates ‘Variability Model’, testing the effect of time, surface and path.**

Intercept = referenced level (day one, straight line, soft surface).

Significance codes: 0 - < 0.001 ‘***’ 0.001 - < 0.01 ‘**’ 0.01 - < 0.05 ‘*’ 0.05 - < 0.1 ‘.’
